# The Lady Chichester Hospital, Brighton

**Published:** 1913-07-26

**Authors:** 


### THE LADY CHICHESTER HOSPITAL, BRIGHTON.

A SPECIAL matinée given at the Court Theatre last May in aid of its Hove Branch brought the Lady Chichester Hospital into public notice. It may be of interest to many to give briefly the history of this institution. Starting in a very small way, about seven years ago, with simply a dispensary for women and children, officered by medical women, the work has developed rapidly, and to-day the Lady Chichester Hospital has three flourishing branches.

First, the Hospital for Nervous Diseases, 70 Brunswick Place, Hove.

Second, the Hospital for Medical and Surgical Cases, 4 and 6 Ditchling Road, Brighton.

Third, the Out-patients' Department, Ditchling Road, at which about 8,000 patients were treated last year.

The hospital is entirely officered by medical women and administered by a committee of women. Its special usefulness lies in the fact that it caters for the large number of women and children who are not eligible for an ordinary free hospital, and yet who cannot afford attendance in a nursing home. Among these are many educated and self-supporting women living in rooms, in which no regular attendance is provided, and in which it is impossible to face a serious illness. They feel perhaps more than any other class the rush and strain of modern life, and it is difficult, often impossible, for them to save any portion of their income with which to meet the inevitable rainy day. To such deserving cases (very many, alas! but little known, for they are very silent about their anxieties) the Lady Chichester Hospital for Women and Children opens its doors.

The branch for nervous diseases is at present the only one of its kind in England, providing, as it does, for all forms of nervous breakdown and borderland cases. A beginning was made in a house in Roundhill Crescent. It contained eleven beds, but this soon proved hopelessly inadequate, and the committee searched for larger quarters. It was difficult to find a house at a moderate rental, with the necessary cheerful surroundings, and in the right position. Finally the council approved of 70 Brunswick Place as meeting these requirements, and in June 1912 the move was made.

Here thirty-five patients can be accommodated. Applications for admission are very numerous, coming from all parts of the kingdom. In the past year, for instance, there have been applications from Dublin, North Wales, North Devon, Oxfordshire, Worcestershire, Middlesex, Hampshire, Kent, and Nottinghamshire, as well as from Sussex. The results are proving very satisfactory, a large proportion of the women and girls returning to their homes after treatment completely cured.

From the beginning of the dispensary the need of an in-patients' hospital was felt, but until some definite help was forthcoming it was impossible to make a start. Last year, however, the council received two very generous offers: one friend promised to pay the rent and rates for three years, and another gave a sum of £200 towards the furnishing of this house. The council decided this would justify them in taking the step forward. A committee was appointed and a start at once made. Two houses, 4 and 6 Ditchling Road, were secured, made to communicate, and altered into a convenient, if small, hospital.

There are two private wards with three or four beds, and also single rooms. In all, twelve patients can be received. The position is good, facing the "Level" and the sea, and the wards and rooms are particularly bright and comfortable. The patients have everything in their favour, not only as regards the cheeriness and comfort of the accommodation, but also in the fact of having the services of a very efficient staff. The fees are exceedingly moderate—7s. 6d. to three guineas weekly, according to the rooms and the state of the patient's purse. The initial expense has been large, but a sum of £500 would pay off all debts incurred and give this branch a good start.

The Out-patients' or Dispensary Department, which is the oldest branch of the work, is carried on in the entire lower part of 4 and 6 Ditchling Road, and contains waiting room, consulting room, dispensing room, etc.

The hours are: 2 to 4 P.M. and 7 to 9 P.M. daily. Here the number of patients is very large—between sixty and seventy on some days. As is the case of the other branches of the hospital, they come not only from Brighton, but from all parts of Sussex, such as Hayward's Heath, Lewes, etc. No letter is needed. Patients are accepted on the merits of the case. A small charge, generally 3d., is made for the medicine, and care is taken to prevent abuse.

